# Radiologic results of additional single screw fixation with lateral locking plate after hybrid closed-wedge high tibial osteotomy

**DOI:** 10.1186/s43019-020-00085-w

**Published:** 2020-12-14

**Authors:** Nobuyuki Hiraoka, Shuji Nakagawa, Eigo Otakara, Hiroaki Inoue, Kenji Takahashi, Yuji Arai

**Affiliations:** 1 Department of Orthopaedics, Graduate School of Medical Science, Kyoto Prefectural University of Medicine, Kyoto, Japan; 2 Department of Orthopaedics, Saiseikai Shiga Hospital, Ritto, Shiga 520-3046 Japan; 3 Department of Sports and Para-Sports Medicine, Graduate School of Medical Science, Kyoto Prefectural University of Medicine, 465 Kajiicho, Kawaramachi-Hirokoji, Kamigyo-ku, Kyoto, 602-8566 Japan

**Keywords:** Bone union, Screw fixation, Hybrid closed-wedge high tibial osteotomy, Knee osteoarthritis, Spontaneous osteonecrosis of the knee, Cannulated cancellous screw, Callus, Locking plate

## Abstract

**Background:**

Hybrid closed-wedge high tibial osteotomy (hybrid CWHTO) is an effective surgical treatment for medial compartment osteoarthritis of the knee. Our study investigated whether the combination of a lateral locking plate and a single medial screw promoted bone union after hybrid CWHTO.

**Methods:**

The study cohort consisted of 30 patients (15 men and 15 women) who underwent hybrid CWHTO for medial compartment osteoarthritis or spontaneous osteonecrosis of the knee. Sixteen knees were fixed with a lateral locking plate (LP group), and 17 were fixed with both a lateral locking plate and a cannulated cancellous screw on the medial side of the tibia (LPS group). The times to bone union, radiolucency, and callus formation at the osteotomy site were evaluated radiographically.

**Results:**

The mean postoperative time to radiographic confirmation of bone union was 5.5 ± 2.6 months in the LP group and 3.4 ± 1.5 months in the LPS group. Radiolucency at the osteotomy site and excess callus formation on the posterior side of the tibia were lower in the LPS group than in the LP group.

**Conclusions:**

This modified hybrid CWHTO combining a lateral locking plate and a cannulated cancellous screw on the medial side of the tibia improves the stability of the osteotomy site and shortens the period of bone union.

## Background

High tibial osteotomy (HTO) is a useful surgical treatment for medial osteoarthritis (OA) of the knee and spontaneous osteonecrosis (ON) of the femur. Two main types of HTO procedures are performed most frequently: lateral closed-wedge HTO, which closes the lateral side of the tibia, and medial open-wedge HTO, which opens the medial side of the tibia [1, 2]. Medial open-wedge HTO has recently shown good clinical outcomes in patients with medial knee OA [3, 4]. However, excess correction of the lower limbs has been associated with various complications, including OA of the patellofemoral joint, increased soft tissue tension, lateral hinge fracture, and delayed bone union [5–7]. Although closed-wedge HTO (CWHTO) can be adapted for knees with advanced varus deformity, this procedure has disadvantages such as leg shortening and lateral offset of the proximal tibia associated with the osteotomy. These drawbacks may be overcome by combining medial open-wedge and lateral CWHTO (hybrid CWHTO) [8].

In hybrid CWHTO, the hinge point is set at two-thirds of the osteotomy line from the lateral side, followed by an oblique osteotomy with biplanar cutting from the lateral part of the proximal tibia. The proximal and distal fragments are then rotated; the lateral side is closed, and the medial side is opened. Internal fixation is performed using a locking plate at the lateral side of the tibia. Compared with conventional CWHTO, this method can achieve greater correction while resecting a smaller bone volume. In addition, the biplanar osteotomy method increases the bone contact area and stabilizes the osteotomy site. Hybrid CWHTO has been reported to be more effective than medial open-wedge HTO in patients with OA of the patellofemoral joint and those with advanced varus knees [9]. When the deformity correction exceeds 10 degrees during open-wedge HTO, the patella height is reduced, increasing the risk of patellofemoral joint degeneration [6]. In hybrid CWHTO, the tibial tuberosity is cut using the biplanar osteotomy method, and the patellar tendon insertion point is elevated anteriorly and repositioned proximally. Pressure on the patellofemoral joint is thereby diminished. Hence, hybrid CWHTO is effective for knees with medial-compartment OA and patellofemoral joint OA [8]. In one study, although bone union was achieved in an average of 4.5 months, 25% of advanced varus knees required more than 6 months for bone union, allowing full weight-bearing within 4 weeks after hybrid CWHTO [10]. Hybrid CWHTO was described as a “no medial bone cortex hinge” procedure requiring an appropriate postoperative program. Improving the fixation of the medial fragment in hybrid CWHTO may be necessary to promote early full weight-bearing and bone union.

Dual plate fixation is sometimes performed on the medial and lateral sides of the tibia to keep the osteotomy site stable. Nevertheless, this method is highly invasive and carries the risk of complications. We hypothesized that using an additional screw on the medial side of the tibia would shorten the period of bone union after hybrid CWHTO. This study aimed to determine the effect of an additional screw fixation on bone healing after hybrid CWHTO.

## Methods

The study protocol was approved by the institutional review board of our hospital (approval number 410). Informed consent was obtained from all participants.

### Patients

This retrospective case-series included 33 knees of 30 consecutive patients (15 knees in 15 men and 18 knees in 15 women) who underwent hybrid CWHTO for medial compartment OA or spontaneous ON of the knee at our hospital. Patients were included if they had symptomatic unicompartmental OA or ON of the medial femorotibial joint. Hybrid CWHTO was performed when the projected correction angle was greater than 11 degrees. Hybrid CWHTO was also indicated in patients with a projected angle of less than 10 degrees when the severity of patellofemoral OA was greater than stage III radiographically or when patients with knee OA experienced anterior knee pain or crepitus [9]. Patients were excluded if they had an infection or inflammatory disease such as rheumatoid arthritis. Patients in the locking plate (LP) group underwent fixation with a lateral locking plate between April 2017 and April 2018, and patients in the locking plate plus screw (LPS) group underwent fixation with a lateral locking plate and a single cannulated cancellous screw (CCS) between May 2018 and July 2019.

### Surgical procedure

A radiograph of the anteroposterior view of the entire leg with the patient in the standing position was used for preoperative planning [8]. All patients underwent initial knee arthroscopy, including debridement of the degenerated meniscus and microfractures and resection of osteophytes. Hybrid CWHTO was performed as described [8]. Briefly, fibular osteotomy at the mid-portion and segmental resection were performed before biplanar osteotomy of the tibia.

Osteotomy of the tibia was performed in the knee extension position. A longitudinal, lateral skin incision was made, and the proximal part of the tibialis anterior muscle and periosteum at the osteotomy site of the tibia were elevated laterally. To determine the proximal osteotomy line, the first Kirschner wire was inserted guided by fluoroscopy. The first Kirschner wire was placed from 35 mm distal to the lateral proximal tibial joint surface to 15 mm distal to the medial proximal tibial joint surface, just distal to the medial collateral ligament (MCL) deep layer attachment site. The second, proximal Kirschner wire was inserted posteriorly parallel to the first wire. The hinge point was set at two-thirds of the osteotomy line from the lateral side, dividing the proximal tibial osteotomy line by approximately 1:2. The third Kirschner wire was inserted toward the hinge point percutaneously from anterior to posterior. To determine the distal osteotomy line, an angle gauge was set at the third Kirschner wire. The fourth, distal Kirschner wire was inserted along the angle gauge from lateral cortex to the hinge point. The fifth Kirschner wire was inserted posteriorly, parallel to the fourth Kirschner wire. The osteotomy was performed along the proximal and distal osteotomy lines from the proximal lateral cortex to the hinge point, using a bone saw and chisel. The separate ascending cut of the biplanar osteotomy was then made behind the patella tendon insertion in the frontal plane, retaining the tibial tuberosity with approximately 10-mm thickness. The lateral closed-wedge bone block between the proximal and distal osteotomy line was carefully removed. Finally, the medial cortex was gently cut along the first osteotomy line using chisels to preserve the superficial layer of the MCL. Thus, the medial soft tissue, including the MCL and periosteum, was not released. After all the osteotomies were completed, the 33% medial side was opened, and the 67% lateral side was closed.

**Fig. 1:**
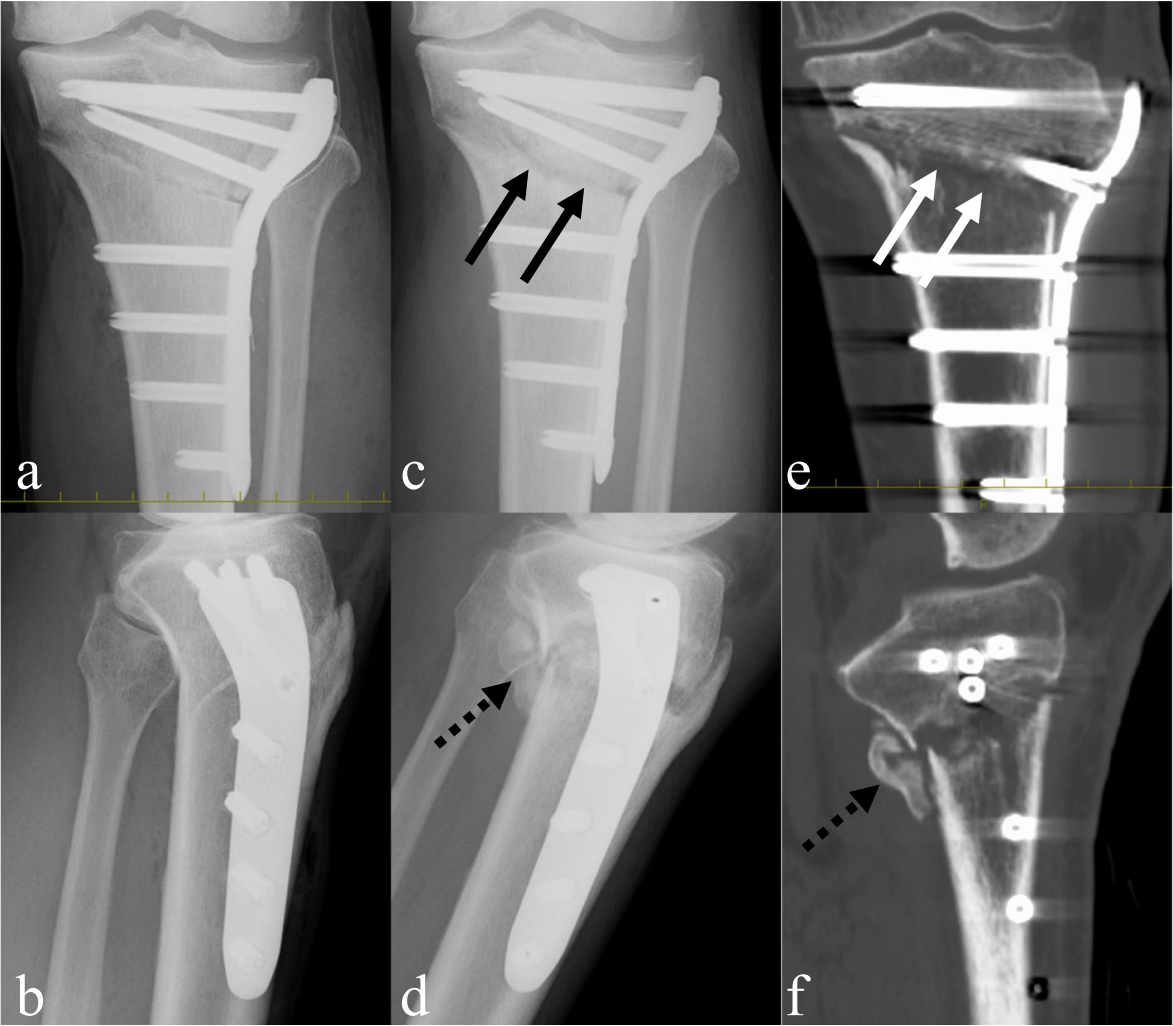
Representative imaging findings in the knees fixed with the lateral locking plate (LP group). **a**, **b** X-ray imaging on postoperative day 0. **c**, **d** X-ray imaging at 3 months. **e**, **f** Computed tomography scans at 3 months. *Solid arrow*, radiolucency at the osteotomy site. No callus. *Dotted arrows*, excess callus formation at the posterior osteotomy site. Discontinuous callus

**Fig. 2:**
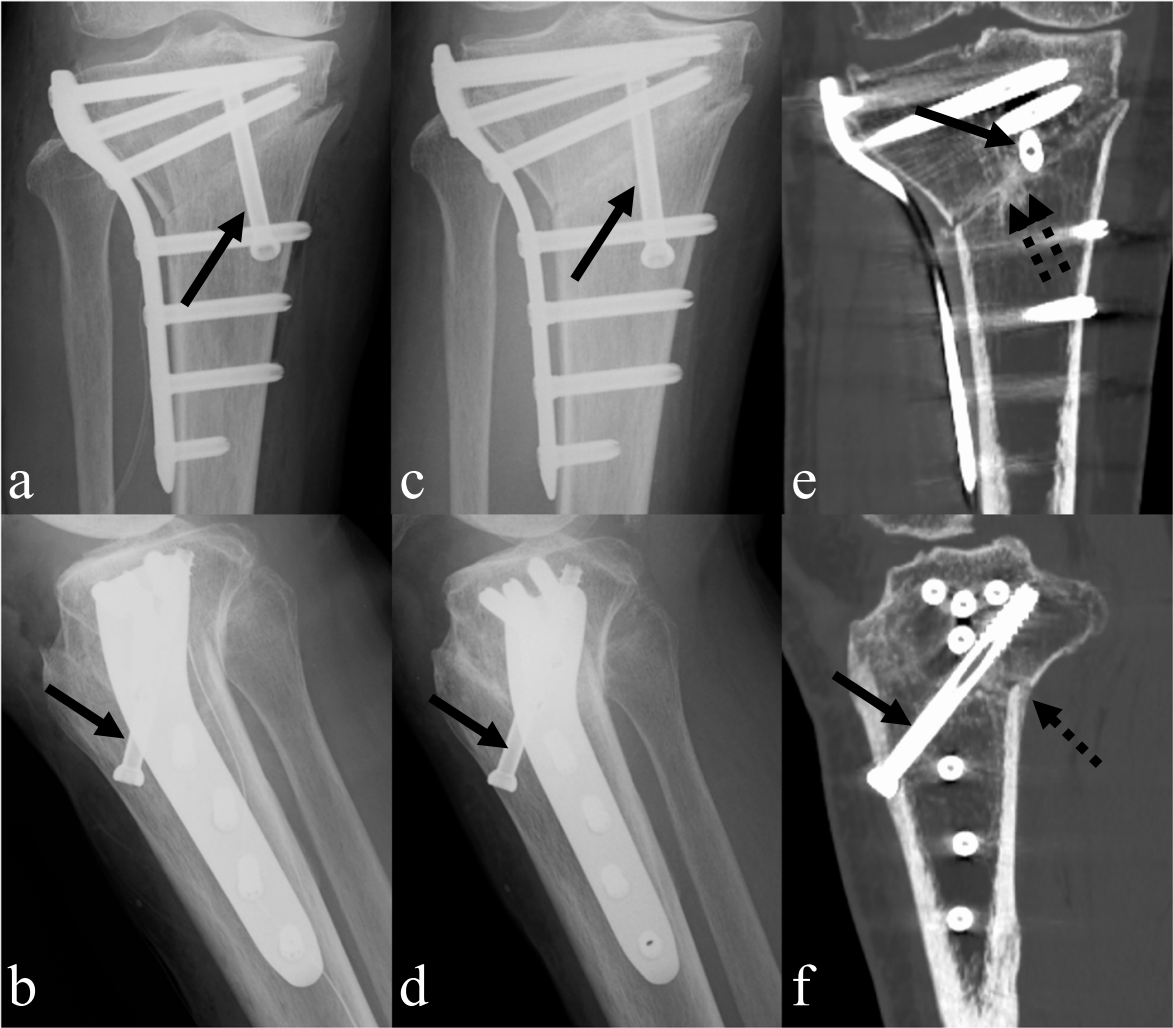
Representative imaging findings in the knees fixed with a lateral locking plate and a cannulated cancellous screw on the medial side of the tibia (LPS group). **a**, **b** X-ray imaging on postoperative day 0. **c**, **d** X-ray imaging at 3 months. **e**, **f** Computed tomography scans at 3 months. *Solid arrow*, a half-thread cannulated cancellous screw. *Dotted arrow*, continuous bridging immature callus. *Double dotted arrow*, continuous remodeled callus

A locking plate (Tris lateral plate system; Olympus Terumo Biomaterials, Tokyo, Japan) was fixed to the proximal fragment using locking screws. With the assistant applying an axial load to both bone fragments, the osteotomy site was compressed using the compression device set between the plate and distal fragment. Locking screws were inserted into the distal bone fragment while confirming correct alignment using fluoroscopy. In the LPS group, a 6.5-mm half-thread CCS (Asnis III; Stryker Limited, Kalamazoo, MI, USA) was added after plate fixation; an additional skin incision was not required for insertion of the CCS. With the knee in an extended position, an anteroposterior view of the proximal tibia was observed with fluoroscopy. A guide pin was inserted from the medial side of the tibial tubercle, 25 mm distal to the hinge point. In the anteroposterior view, the guide pin was directed to the posterior cruciate ligament (PCL) attachment via the exterior of the hinge point. Next, by observing the lateral view with the knee joint in the flexed position, we confirmed that the guide pin was inserted to the PCL attachment through the posterior part of the screw of the lateral locking plate. The angle between the guide pin and the tibial axis was approximately 50 degrees. After creating a countersink to prevent screw head protrusion, a half-thread CCS was inserted (Figs. 1 and 2).

### Postoperative rehabilitation

The postoperative rehabilitation regimen was the same for both groups. Active and passive range-of-motion and isotonic muscle-strengthening exercises were started the day after surgery. After 1 week, patients began partial weight-bearing supported by a walker. After 2 weeks, full weight-bearing walking was allowed depending on the pain.

### Radiological measurements and follow-up evaluation

Standard radiography was performed 1 month before the hybrid CWHTO. Postoperative radiography was performed every month on every patient. Computed tomography (CT) scans were performed 3 months after surgery (7 patients in the LP group, 11 patients in the LPS group). Coronal knee alignment was evaluated by measuring the percentage of mechanical axis (%MA), the femorotibial angle (FTA; defined as the lateral angle between the center line of the femur and the tibia on coronal radiographs in the standing position), the medial proximal tibial angle (MPTA), and the mechanical lateral distal femoral angle (mLDFA).

The time to bone union was determined by radiographic imaging, as described [10]. Radiolucency at the osteotomy site was defined as the reduction in bone density in the area from the lateral side of the osteotomy to the hinge point on frontal X-ray images compared to that on the day of surgery.

The term ‘callus’ is used to describe the calcified tissue around the osteotomy site [11]. The osteotomy sites were investigated using a Tomographic Union Score (TUS) based on the callus appearance on the CT, as previously described [12]. The score at the fracture edges was defined as follows: score 1, no callus (Fig. 1e); score 2, discontinuous callus (Fig. 1f); score 3, continuous bridging immature callus (Fig. 2f); score 4, continuous remodeled callus (Fig. 2f). Continuous bridging immature callus and continuous remodeled callus were treated as bone union. The anterior flange and posterior site were evaluated using sagittal images, and the medial, hinge point, and lateral sites were evaluated using coronal images.

Callus formation was defined as excess if the callus was > 10 mm long and > 5 mm wide on lateral X-ray images and sagittal CT images. The time to bone union on radiography, the presence or absence of radiolucency, excess callus formation, and bone union on CT were identified based on the agreement of the two orthopedic surgeons (HN and OE) specializing in knees, with 18 and 16 years of experience, respectively.

At the final follow-up, the knees were assessed using the Japanese Orthopedic Association (JOA) scoring system [13], and MPTA was also measured [14].

### Statistical analysis

Data are expressed as means ± standard deviations and were analyzed using EZR (Saitama Medical Center, Jichi Medical University), a graphical user interface for R (The R Foundation for Statistical Computing, version 2.13.0). Differences between the LP and LPS groups in terms of patient characteristics and radiographically measured parameters were analyzed using the Student’s *t*-test. Differences in radiolucency and excess callus formation were analyzed using the chi-square test. In all analyses, *p* < 0.05 was defined as statistically significant. The total sample size (*n* = 33) provided more than 80% power to detect a difference in the time to bone union (months) between the LP and LPS groups (α = 0.05, β = 0.20).

## Results

The 30 patients included 15 men and 15 women of median age 66 years (range 39–79 years), median height 158 cm (range 148–178 cm), and median body mass index (BMI) 25.0 kg/m^2^ (range 18.2–33.9 kg/m^2^). Of the 33 knees, 16 underwent LP fixation and 17 underwent LPS fixation. There were no significant differences between the two groups in terms of baseline demographic or clinical characteristics (Table 1). Preoperative and postoperative %MA, FTA, MPTA, and mLDFA of hybrid CWHTO patients did not differ significantly between the two groups (Table 2). All patients achieved bone union within 12 months after surgery.

**Table 1:** Patient characteristics

	LP group	LPS group	*P*-value
Number of knees	16	17	
Male / female	9 / 7	7 / 10	
Age (Years)	61.4 ± 10.4	67.3 ± 8.7	n.s.
Height (m)	160.9 ± 8.0	159.1 ± 8.9	n.s.
Body Weight (Kg)	67.2 ± 12.6	65.1 ± 3.4	n.s.
Body mass index (kg/m^2^)	25.9 ± 4.2	25.7 ± 4.4	n.s.
surgical time (minutes)	135.7 ± 14.1	139.6 ± 20.2	n.s.
smoking history	3	6	n.s.

*Abbreviations*: *LP* locking plate, *LPS* locking plate and a cannulated cancellous screw, *n.s.* not significant

**Table 2:** Changes in knee alignment after hybrid closed-wedge high tibial osteotomy (hybrid CWHTO)

	LP group	LPS group	*P*-value
%MA (%)			
Pre-op	16.4 ± 10.4	13.5 ± 15.6	n.s.
Post-op	64.2 ± 11.5	64.1 ± 13.4	n.s.
FTA (degrees)			
Pre-op	181.8 ± 2.1	182.6 ± 3.3	n.s.
Post-op	169.6 ± 3.3	170.5 ± 3.2	n.s.
MPTA (degrees)			
Pre-op	83.0 ± 2.7	83.4 ± 2.6	n.s.
Post-op	94.0 ± 2.3	94.5 ± 3.2	n.s.
mLDFA (degrees)			
Pre-op	87.4 ± 1.4	88.6 ± 2.1	n.s.

*Abbreviations*: *FTA* femorotibial angle, *LP* locking plate, *LPS* locking plate and a cannulated cancellous screw, *MA* mechanical axis, *mLDFA* mechanical lateral distal femoral angle, *MPTA* medial proximal tibial angle, *n.s* not significant

The mean time to radiographic confirmation of bone union was significantly longer in the LP group (5.5 months) than in the LPS group (3.4 months). The difference in mean surgical time between the groups was not significant (LPS group, 139.6 ± 20.2 min; LP group, 135.7 ± 14.1 min). Only one male patient in the LP group experienced a complication (plate breakage 3 months after surgery). A second surgery was performed for this patient, using medial and lateral locking plates, resulting in bone union 7 months after the initial surgery. No other patient experienced any complications, including infections, pseudarthroses, neurovascular injuries, or irritation by the CCS.

**Fig. 3:**
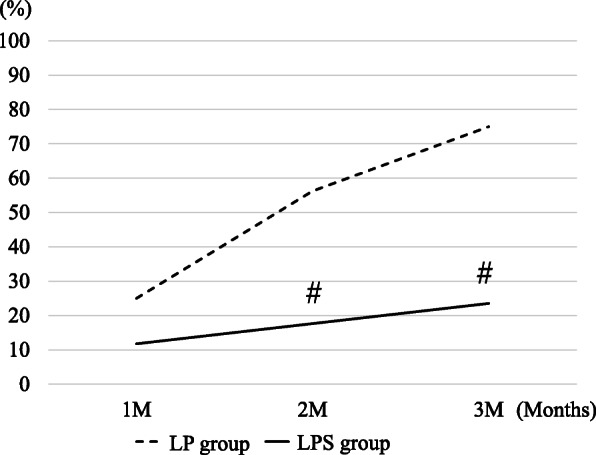
Radiolucency at the osteotomy site. *LP* locking plate, *LPS* locking plate and a cannulated cancellous screw

**Fig. 4:**
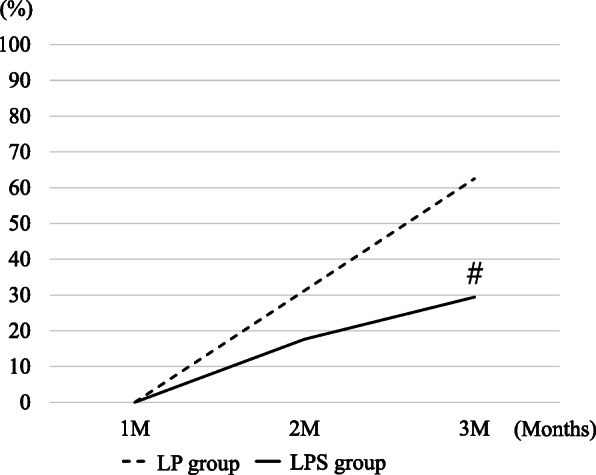
Excess callus formation at the posterior osteotomy site on X-ray image. *LP* locking plate, *LPS* locking plate and a cannulated cancellous screw

The rates of radiolucency at the osteotomy site in the LP and LPS groups were 25.0% and 11.8%, respectively, after 1 month; 56.3% and 17.6%, respectively, after 2 months; and 75.0% and 23.5%, respectively, after 3 months (*p* < 0.05; Fig. 3). The rates of excess callus formation at the posterior osteotomy sites in the LP and LPS groups were both 0% at 1 month; 31.3% and 17.6%, respectively, at 2 months; and 62.5% and 29.4%, respectively, at 3 months (*p* < 0.05; Fig. 4).

Excessive callus formation at the posterior osteotomy site was more common in the LP group on CT imaging performed 3 months after surgery (Table 3). In the LP group, the union rate on CT imaging was less than 50% in all five sites. In the LPS group, however, the union rate was greater than 50% in all sites but the lateral.

**Table 3:** Excess callus formation at the posterior osteotomy site and bone union on Computed tomography scans at 3 months

		LP group	LPS group
Excess callus formation at posterior osteotomy site (%)		85.7	36.4
Bone union	anterior flange (%)	42.9	72.7
	posterior (%)	42.9	63.6
	medial (%)	14.3	54.5
	hinge (%)	14.3	72.7
	lateral (%)	14.3	27.3

The mean follow-up period was 35.9 ± 4.0 months in the LP group and 24.1 ± 4.5 months in the LPS group. The JOA score significantly improved from 67.0 ± 4.9 to 90.3 ± 6.9 in the LP group and from 64.1 ± 5.4 to 90.0 ± 8.7 in the LPS group. There was no significant difference between the two groups.

The MPTA in the LP group was 94.0 ± 2.3 degrees postoperatively and 93.9 ± 2.0 degrees at the final follow-up. The MPTA in the LPS group was 94.5 ± 3.2 degrees postoperatively and 94.1 ± 2.4 degrees at the final follow-up. There was no significant difference between the two groups.

## Discussion

The most important finding of this study was that the mean time to bone union was significantly shorter in the LPS group than in the LP group. Radiolucency of the osteotomy site and excess callus formation on the posterior side of the tibia were lower in the LPS group than in the LP group. These results suggest that an additional CCS near the hinge point can provide minimally invasive and firm fixation of the osteotomy site and can shorten the bone healing period.

In hybrid CWHTO, the osteotomy site is kept firm because of the combination of the lateral locking plate and the increased tension of the superficial bundle of the MCL, both of which lead to improved stability of the medial and lateral compartments [8]. Good clinical outcomes have been reported in patients undergoing hybrid CWHTO for medial knee OA [14]. In CWHTO, nonunion is rare, and delayed union has been observed in 4–8.5% of knees [15–17]. The average period to bone union in hybrid CWHTO was 4.5 months with no patient showing nonunion, although bone union in 25% of patients required more than 6 months [10]. In the present study, the mean time to bone union in the LP group was 5.5 months, with 31% of these patients requiring more than 6 months. Time to bone union, improvement of pain, and gait function recovery may be longer in some patients undergoing hybrid CWHTO with only a unilateral locking plate.

In hybrid CWHTO, the lateral part of the osteotomy site is stabilized due to the contact between the proximal and distal fragments and the lateral locking plate. By contrast, the medial part of the osteotomy site and the tension of the superficial bundle MCL may not provide as much support as the lateral part. In the sagittal plane, the supportability of the anterior part and conditions for bone union were better than those of the posterior part due to the large contact area and the presence of an anterior flange during biplanar osteotomy. The double-plate construct was significantly stiffer than a single locking-plate system in the biomechanical stability of the extra-articular proximal tibial fracture [18]. These findings suggest that the overall stability of osteotomy may be improved if an additional plate is inserted during the hybrid CWHTO procedure. However, placement of a dual plate is associated with significant surgical invasion and increased risk of infection. By contrast, in the present study, insertion of a single CCS without an additional skin incision gave a significant reduction in average time to bone union (3.4 months in the LPS group), with only 6% of patients requiring more than 6 months. Our LPS patients had no complications; therefore, this method was considered safe and effective for the hybrid CWHTO procedure.

In the process of fracture healing, under completely stable fixation, there is minimal visible callus formation or none at all. A gradual disappearance of the fracture line with trabeculae growing across this line is a good sign, while a widening of the gap is a sign of instability. The progression of bone fusion is determined by the absence of radiographic signs of irritation, such as bone resorption or the formation of a cloudy ‘irritation’ callus [19]. When the fracture site is mechanically stable, there is little callus formation. If the site is unstable, excess callus formation will occur; however, a bridging callus will not be formed [20]. Furthermore, Schell et al. [21] reported that semi-rigid fixation resulted in the formation of a larger callus than rigid fixation. Radiolucency at the osteotomy site and excess callus formation at the posterior osteotomy site indicate some instability. Although all patients in this study achieved complete bone union, the rates of radiolucency at 2 and 3 months were significantly higher in the LP group than in the LPS group, and the rates of excess callus formation at 3 months was significantly higher in the LP group than in the LPS group. For some patients in the LP group, the osteotomy site may not have been stable enough. In the LPS group, a single CCS was inserted from the distal fragment to the proximal fragment on the near side of the PCL attachment via the exterior of the hinge point. Because hybrid CWHTO is an oblique osteotomy, there will be a shear force to the osteotomy site under load. The newly formed callus is vulnerable to shear forces, whereas axial traction and pressure promote matrix formation [22]. The CCS may improve the stability of the hinge point and posterior part, thus suppressing the shear force and promoting bone union. Earlier full weight-bearing after open-wedge HTO resulted in earlier clinical improvement [23]. Late limb loading can cause muscle loss and delayed social reintegration. Even in hybrid HTOs, clinical outcomes may be improved if full loading can be achieved early with locking plates and CCS fixation.

This study has limitations. It included relatively few patients. Therefore, there is a need for additional studies assessing larger sample sizes with long-term results, including patient-based outcomes. Furthermore, it was a retrospective design and included few cases for CT evaluation. Nonetheless, this study presents the first clinical results of this new surgical procedure.

## Conclusions

This modified hybrid CWHTO combining a lateral locking plate and a cannulated cancellous screw on the medial side of the tibia improves the stability of the osteotomy site and shortens the period of bone union.

## Data Availability

All data generated or analyzed during this study are included in this published article.

## Abbreviations

BMI: Body mass index
CCS: Cannulated cancellous screw
CT: Computed tomography
CWHTO: Closed-wedge high tibial osteotomy
FTA: Femorotibial angle
HTO: High tibial osteotomy
JOA: Japanese Orthopedic Association
MCL: Medial collateral ligament
mLDFA: Mechanical lateral distal femoral angle
MPTA: Medial proximal tibial angle
OA: Osteoarthritis
ON: Osteonecrosis
%MA: Percentage of mechanical axis
PCL: Posterior cruciate ligament
